# Persistence of systemic and cerebral perfusion impairment in patients with neurocardiac injury after aneurysmal subarachnoid hemorrhage

**DOI:** 10.1186/2197-425X-3-S1-A777

**Published:** 2015-10-01

**Authors:** M Hravnak, KM Yousef, Y Chang, E Crago, RM Friedlander

**Affiliations:** University of Pittsburgh, School of Nursing, Pittsburgh, United States; University of Pittsburgh, School of Medicine, Pittsburgh, Pennsylvania United States

## Introduction

Although it has been demonstrated previously that patients with aneurysmal subarachnoid hemorrhage (aSAH) who also experience neurocardiac injury have lower short term (0-3 days after aneurysm rupture) systemic and cerebral perfusion parameters compared to those without neurocardiac injury, it remains unknown if these changes persist over a longer time.

## Objectives

We aimed to determine if systemic and cerebral perfusion parameters across days 0-14 after SAH were persistently lower when cardiac troponin I (CTnI) was elevated (greater than or equal to 0.3 ng/ml [cTnI-High]) compared to when cTnI was nondetectable (less than 0.01 ng/ml [cTnI-ND]).

## Methods

Longitudinal prospective analysis of 38 patients with aSAH. Inclusion: age 21-75 years, spontaneous aneurysm rupture, Fisher grade >1 and/or Hunt and Hess grade (HH) >2. Exclusion: traumatic SAH, recent myocardial dysfunction. Daily averages of systemic (systolic blood pressure [SBP], diastolic blood pressure [DBP], mean arterial blood pressure [MAP], heart rate [HR]) and cerebral (cerebral perfusion pressure [CPP]) perfusion parameters were used. Blood pressure was measured with arterial line, or if no arterial line then sphygmomanometer. Intracranial pressure (ICP) was measured using external ventricular drain and was used to calculate CPP (MAP-ICP). Mixed model linear regression was performed to test the difference in perfusion over time. Modeling was performed using the daily average for each perfusion parameter for each of days 0 to 14, and both without and with covariates (age, gender HH grade). The independent variable was daily average cTnI-High.

## Results

**Figure 1:**
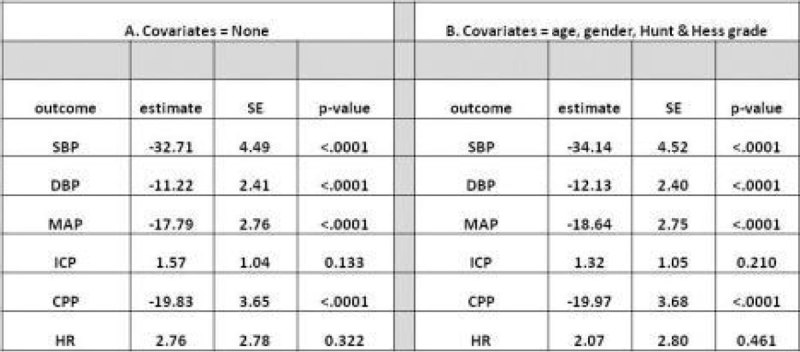
**Mixed Model Linear Regression modelling results using daily average perfusion parameters over days 0 to 14 after subarachnoid haemorrhage without (A) and with (B) covariates. The independent variable is daily average cTnl level > = 0.3 ng/ml.**

Patients were predominantly female (76%) with HH grade 1-2 (61%) and mean age 53 ± 11 years. The mixed model linear regression revealed that cTnI-High was significantly associated with lower SBP, DBP, MAP and CPP compared to cTnI-ND (Figure 1.) when no covariates were accounted for (A), and the significance was maintained when covariates were included (B). ICP and HR were not significantly associated with cTnI-High.

## Conclusions

Elevated levels of cTnI are associated with lower systemic and cerebral perfusion parameters across fourteen days following aSAH. The driving force for the CPP direction was lower blood pressure and not higher ICP. Therefore, neurocardiac injury is associated with persistently lowered perfusion over time, not just within the first few days after aneurysm rupture. Further research is needed to determine if these persistently lower perfusion pressures translate into worse physical and neurocognitive function in the long term, and if more targeted perfusion support is needed for aSAH patients with neurocardiac injury during hospitalization.

## Grant Acknowledgment

NIH NINR R01NR014221

